# Supplementary material for: An accurate and robust imputation method scImpute for single-cell RNA-seq data
Source: Nat Commun. 2018 Mar 8;9:997. doi: 10.1038/s41467-018-03405-7 (PMC5843666; doi:10.1038/s41467-018-03405-7)
Supplement: Supplementary file 1 — Supplementary Information [file 41467_2018_3405_MOESM1_ESM.pdf]

An accurate and robust imputation method scImpute for single-cell RNA-seq data

Li et el.

Supplementary Figures

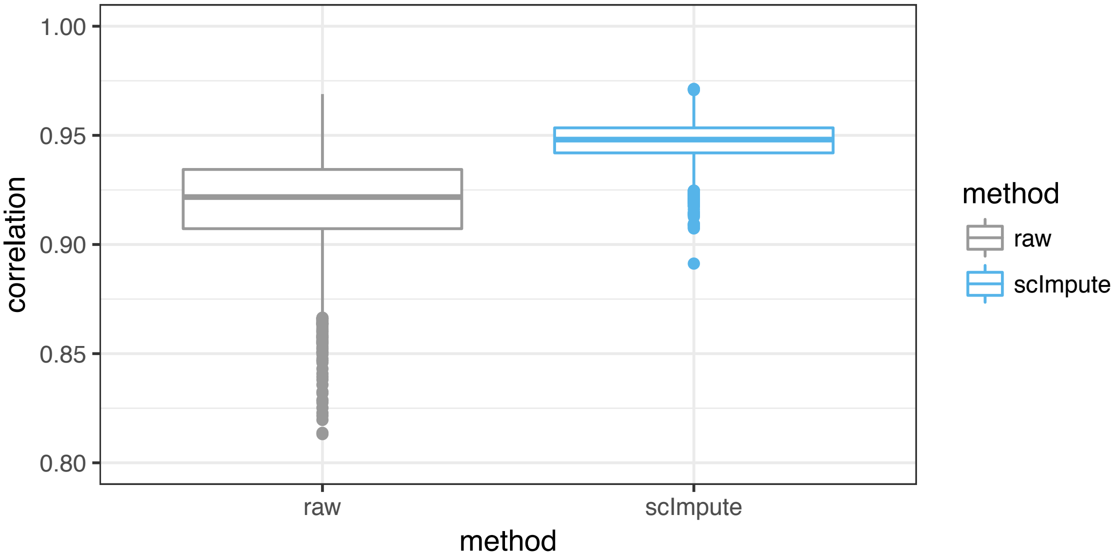

Supplementary Figure 1. Correlations between ERCC spike-ins' counts and their true concentration. The two distributions show the correlations between the ERCC spike-ins' log10(count+1) and log 10(concentration) in the 3,005 mouse cortex cells (one correlation per cell for raw counts or counts corrected by scImpute).

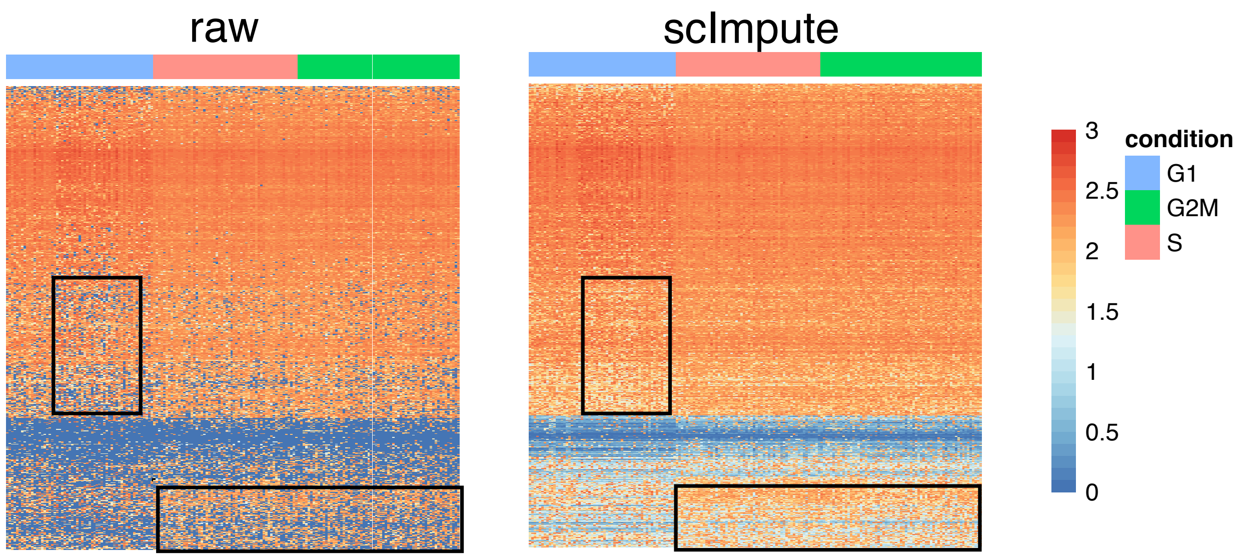

Supplementary Figure 2. Heatmaps showing the log10(count+1) of the 892 cell cycle genes before and after imputation. Rows correspond to genes, and columns correspond to cells.

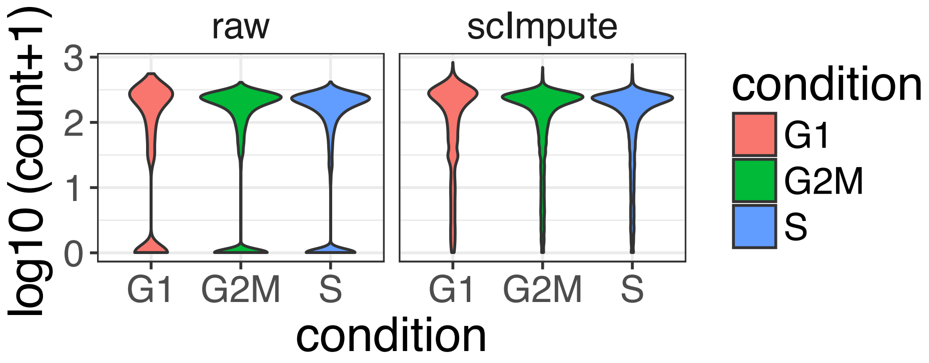

Supplementary Figure 3. Expression of cell cycle genes before and after imputation. Violin plots showing the log10(count+1) of the 892 cell cycle genes in the three phases (G1, G2M, and S). This comparison result shows that scImpute has successfully imputed the dropout expression values of cell cycle genes.

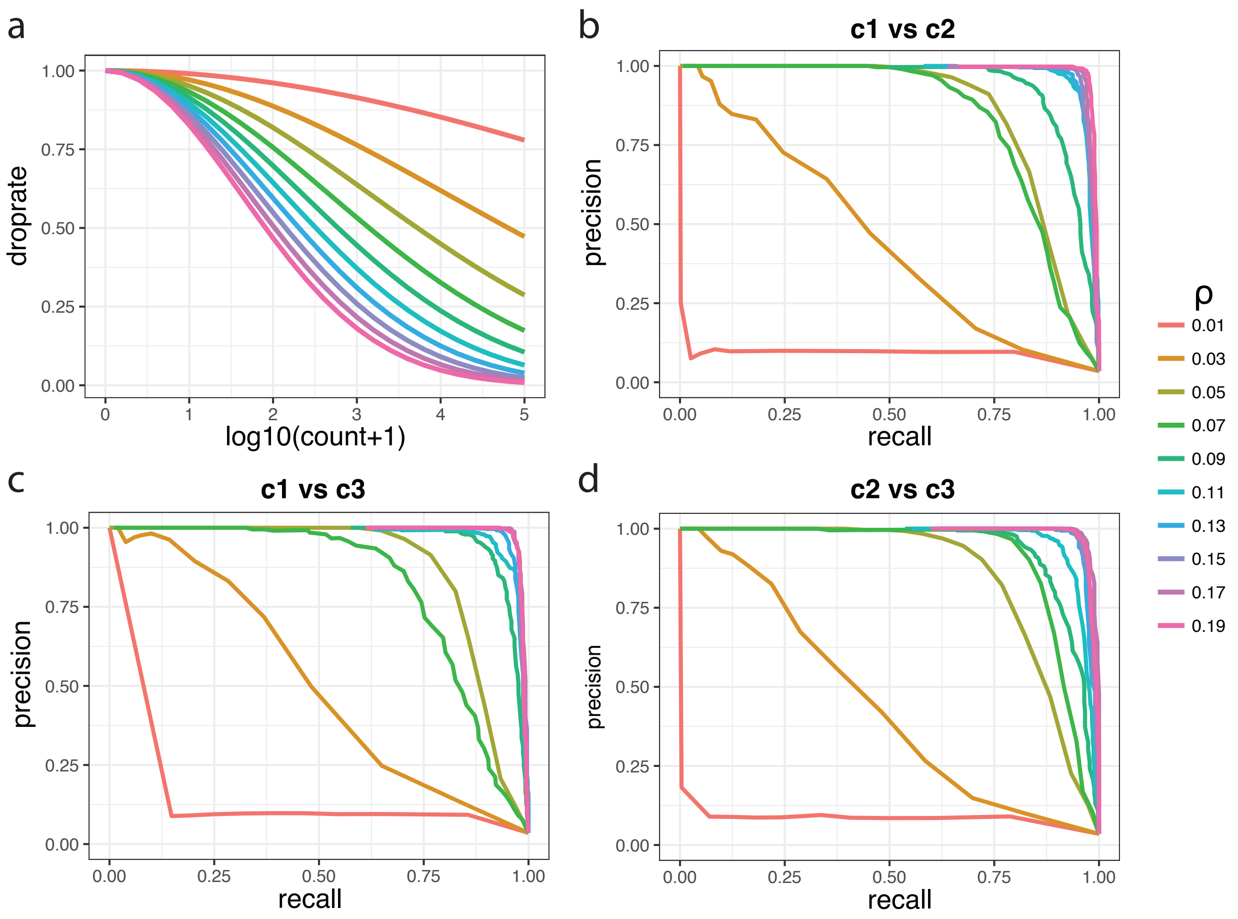

Supplementary Figure 4. Performance of scImpute given different dropout rates in raw simulated data. a: The theoretical dropout rates determined by the double exponential function $exp\ (-\rho \times log_{10}(count+1)^2)$, with ρ varying from 0.01 to 0.19 by a step size of 0.02. **b-d**: The precision-recall curves for the identification of differentially expressed genes from the imputed data.

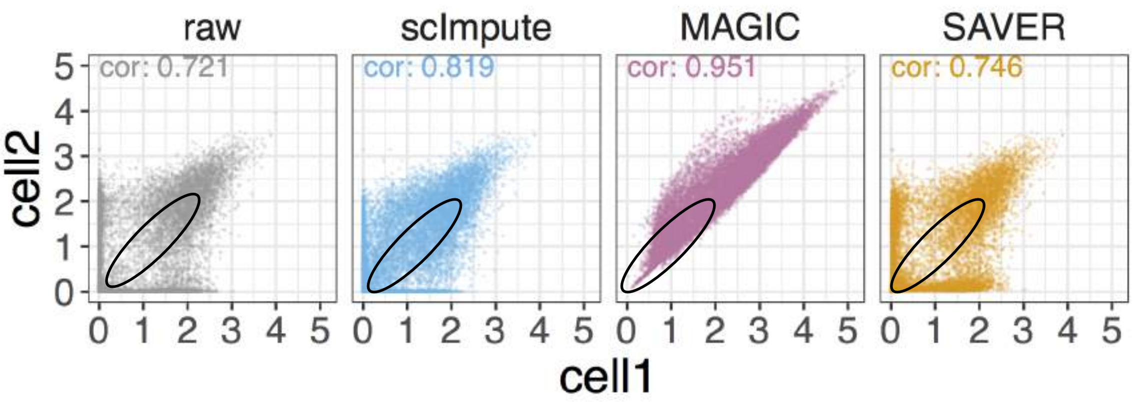

Supplementary Figure 5. The raw and imputed gene expression levels of two mouse embryonic cells of the 16-cell stage. Correlations between the two cells are marked on the top-left of each scatter plot.

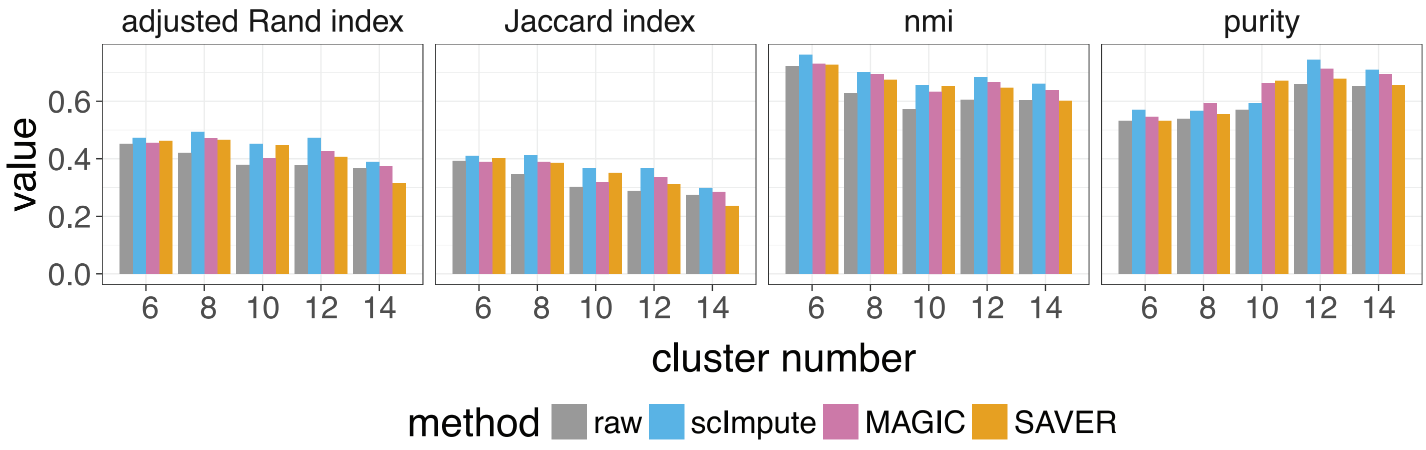

Supplementary Figure 6. The adjusted Rand index, Jaccard index, nmi, and purity scores of clustering results based on the raw and imputed data. Clustering is performed by the spectral clustering algorithm [23] on the single cells' scores in the first two principal components.

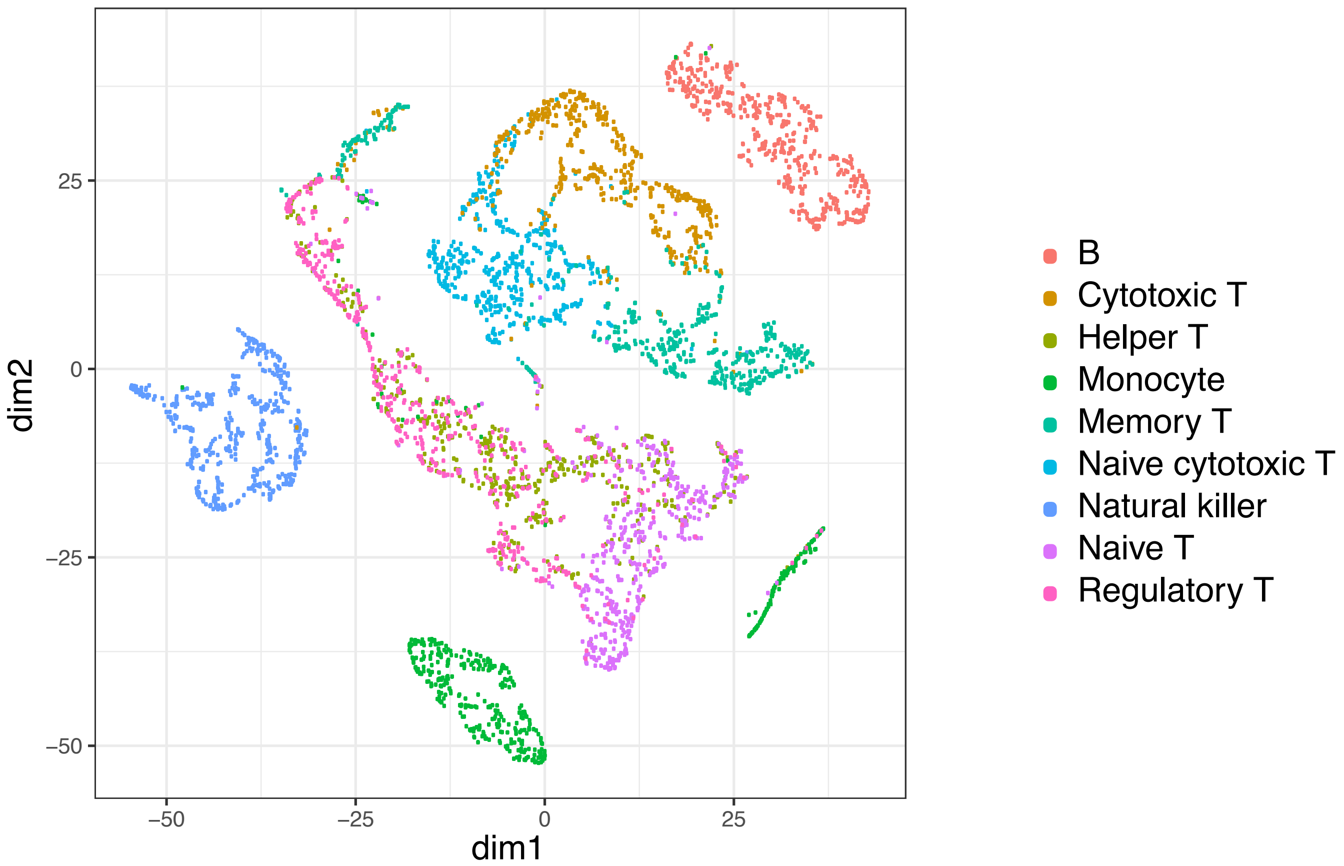

Supplementary Figure 7. The first two dimensions of the t-SNE results calculated from imputed PBMC data by MAGIC.

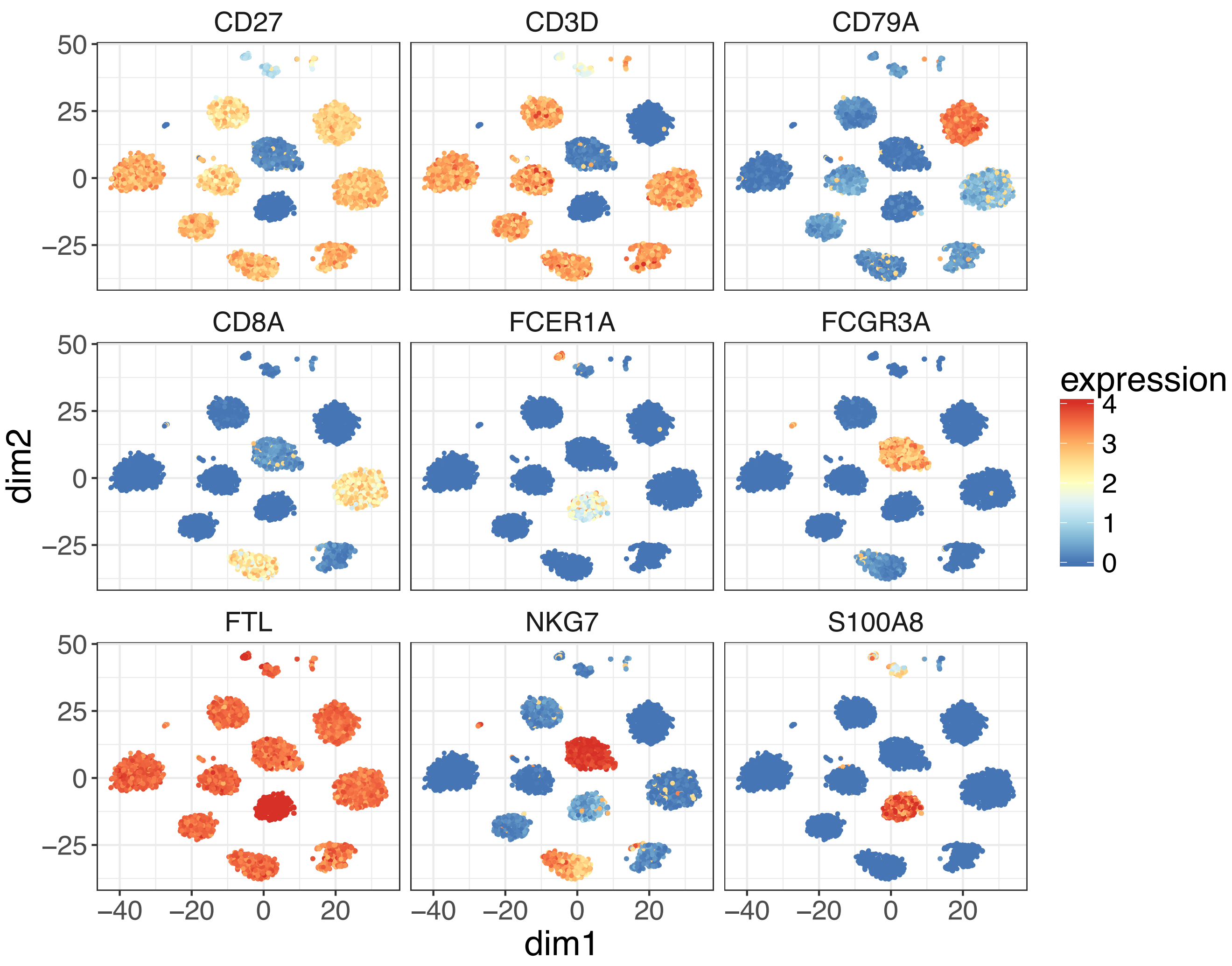

Supplementary Figure 8. Expression levels of marker genes after imputation. The $\log_{10}$ expression levels of nine known marker genes shown in clusters in the first two dimensions of the t-SNE results calculated from imputed PBMC data by scImpute.

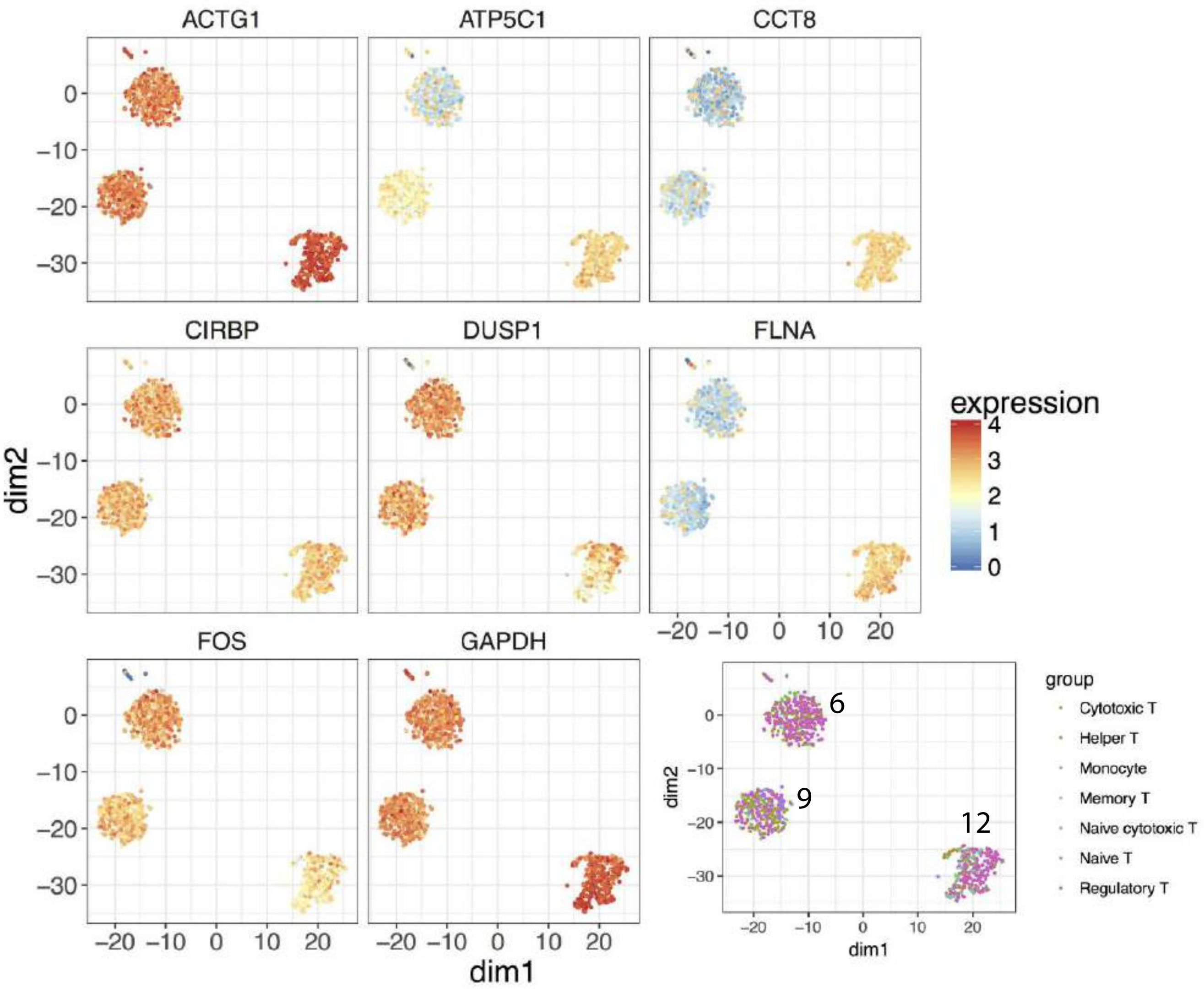

Supplementary Figure 9. Potential marker genes of cell subpopulations. The $\log_{10}$ expression levels of eight potential marker genes to distinguish subpopulations of T cells, which are shown in the first two dimensions of the t-SNE results calculated from imputed PBMC data by scImpute.

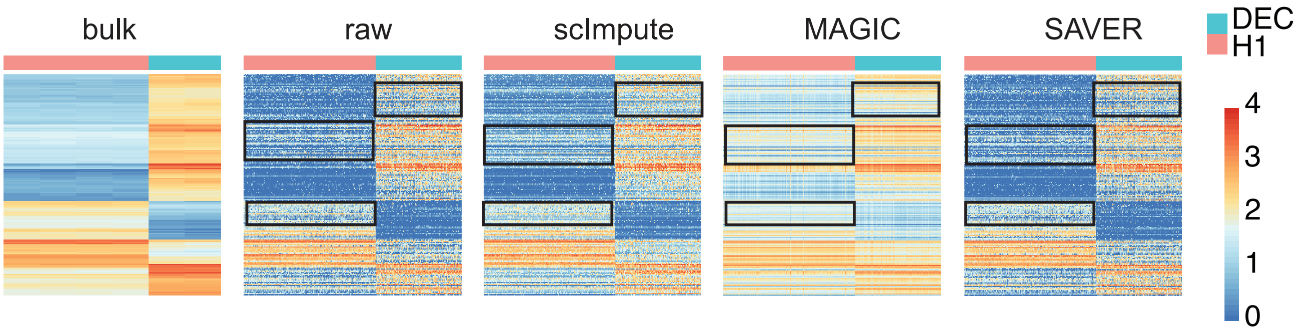

Supplementary Figure 10. Expression levels of differentially expressed (DE) genes. The log_{10} expression profiles of the top 200 DE genes detected in the bulk data by DESeq2 [31].

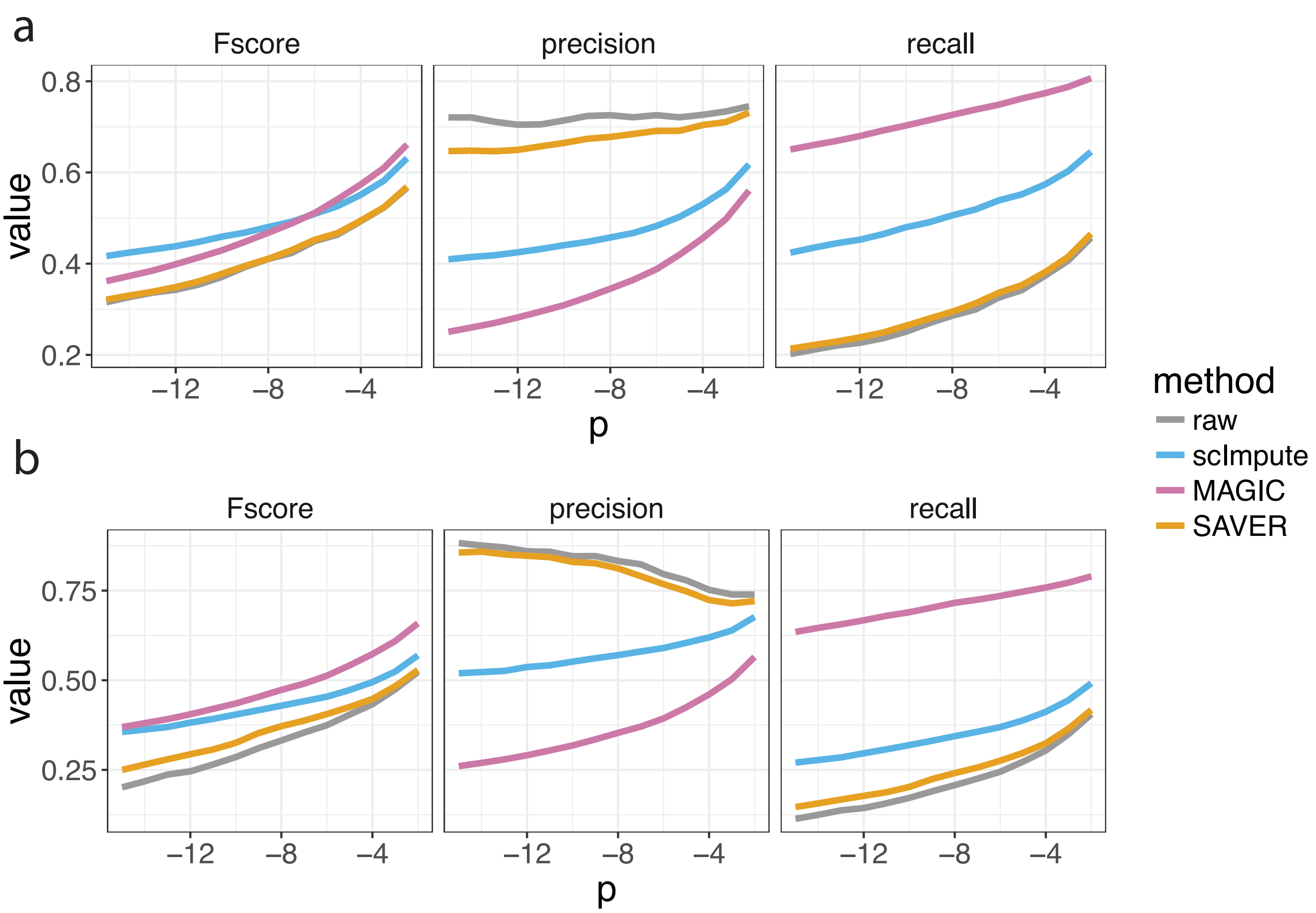

Supplementary Figure 11. Comparison of DE analysis between bulk and single-cell data. a: p-values for both bulk and single-cell data are calculated using DESeq2 [31]. **b**: p-values for bulk data are calculated using DESeq2 and p-values for single-cell data are calculated using MAST [32].

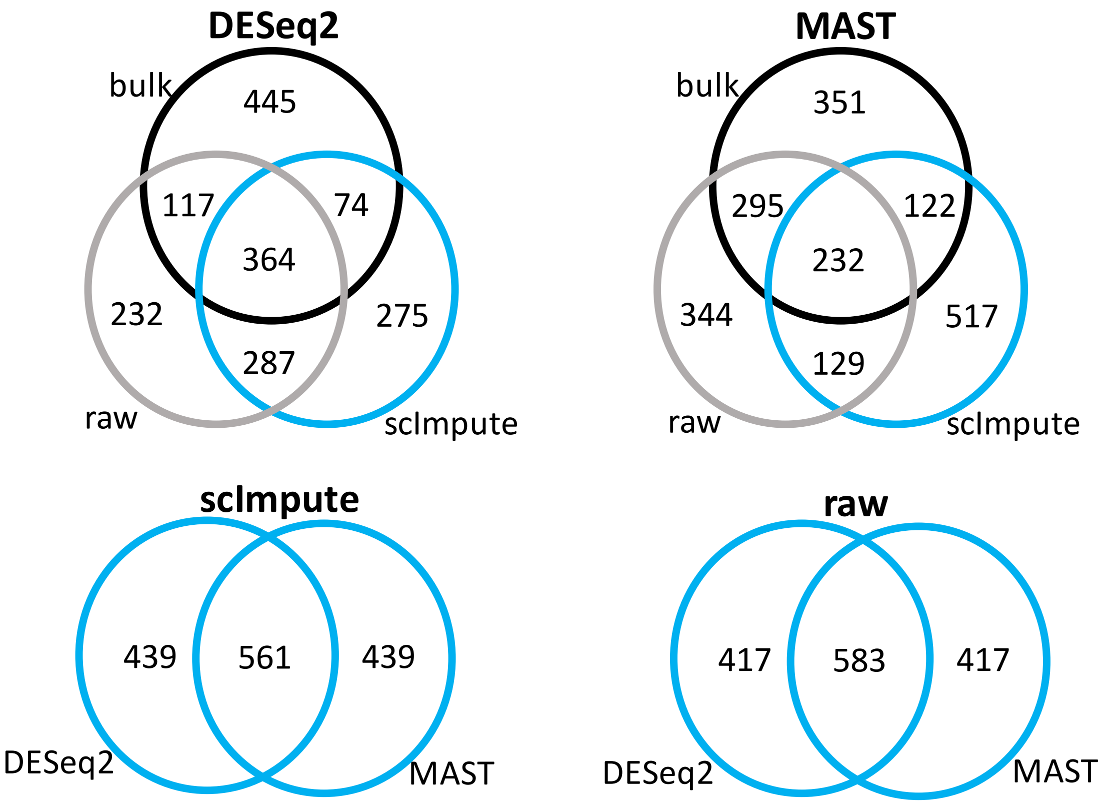

Supplementary Figure 12. Intersection of the top 1,000 differentially expressed (DE) genes detected in bulk, raw, and imputed data by DESeq or MAST. a: DE genes are detected using DESeq2 [31]. **b**: DE genes in bulk data are detected using DESeq2; DE genes in single-cell data are detected using MAST [32]. **c**: Top 1,000 genes detected in scImpute's imputed data by DESeq2 and MAST have 561 in common. **d**: Top 1,000 genes detected in raw data by DESeq2 and MAST have 583 in common.

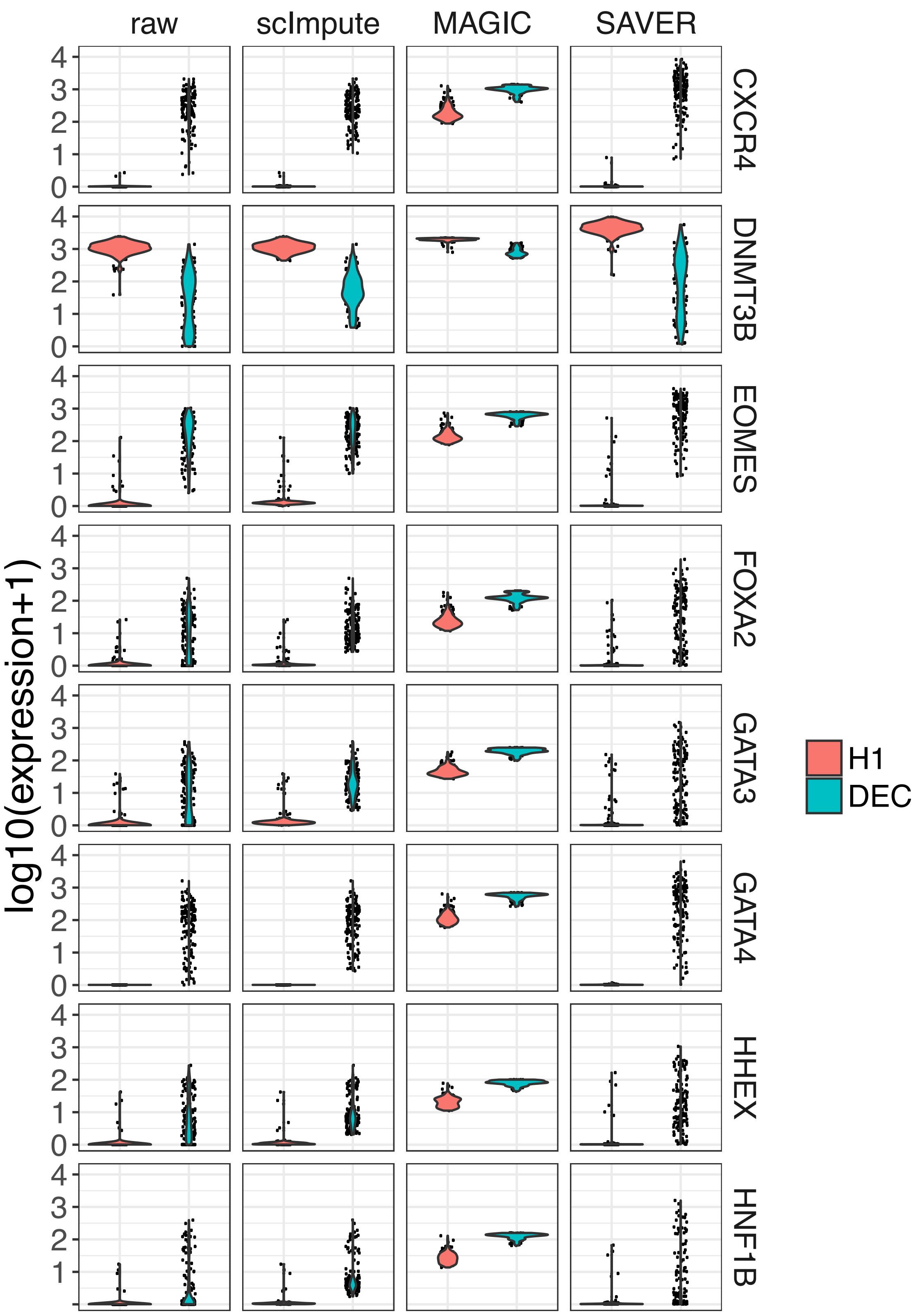

Supplementary Figure 13. Violin plots showing $\log_{10}$ expression levels of nine genes before and after imputation.

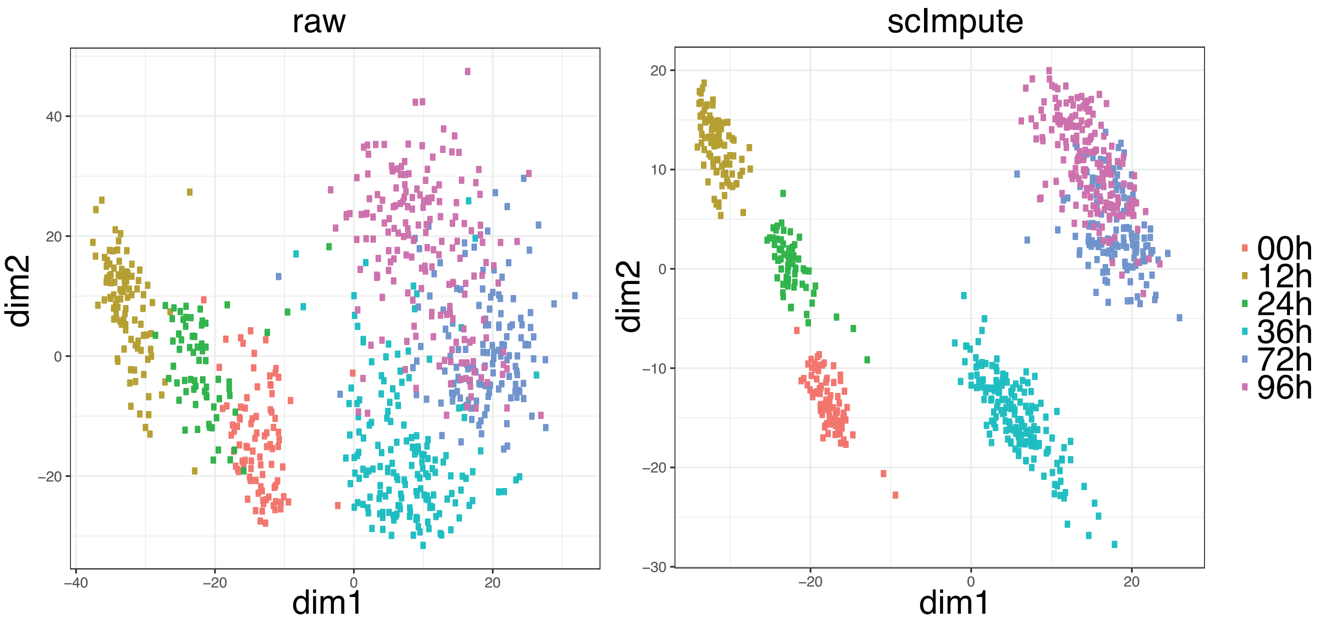

Supplementary Figure 14. The first two dimensions of principal component analysis (PCA) results calculate from raw and imputed time-course ESC data.

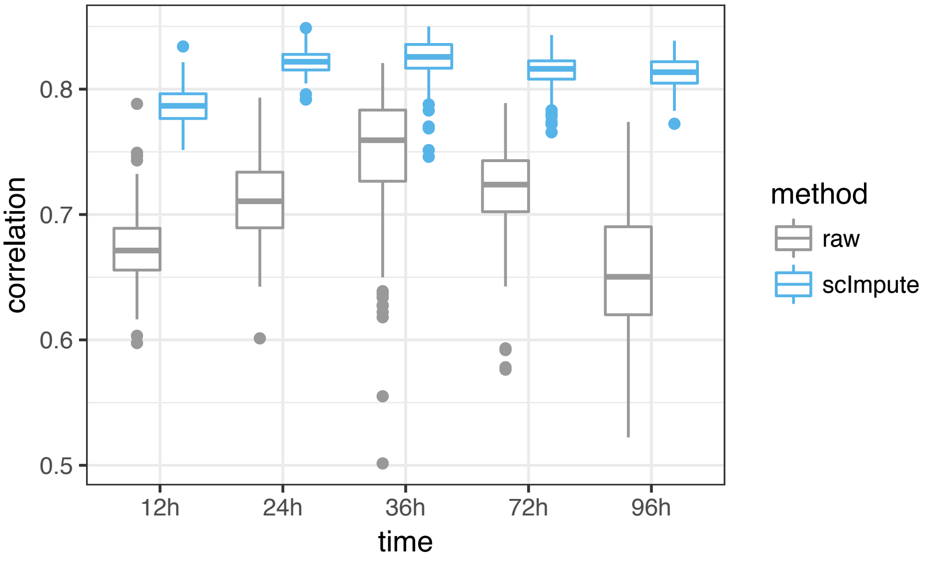

Supplementary Figure 15. Correlations between gene expression in single-cell and bulk data. The Pearson correlation coefficients are calculated between each individual cell and averaged bulk data, at each time point. The correlations based on the imputed data are significantly higher than those based on the raw data.

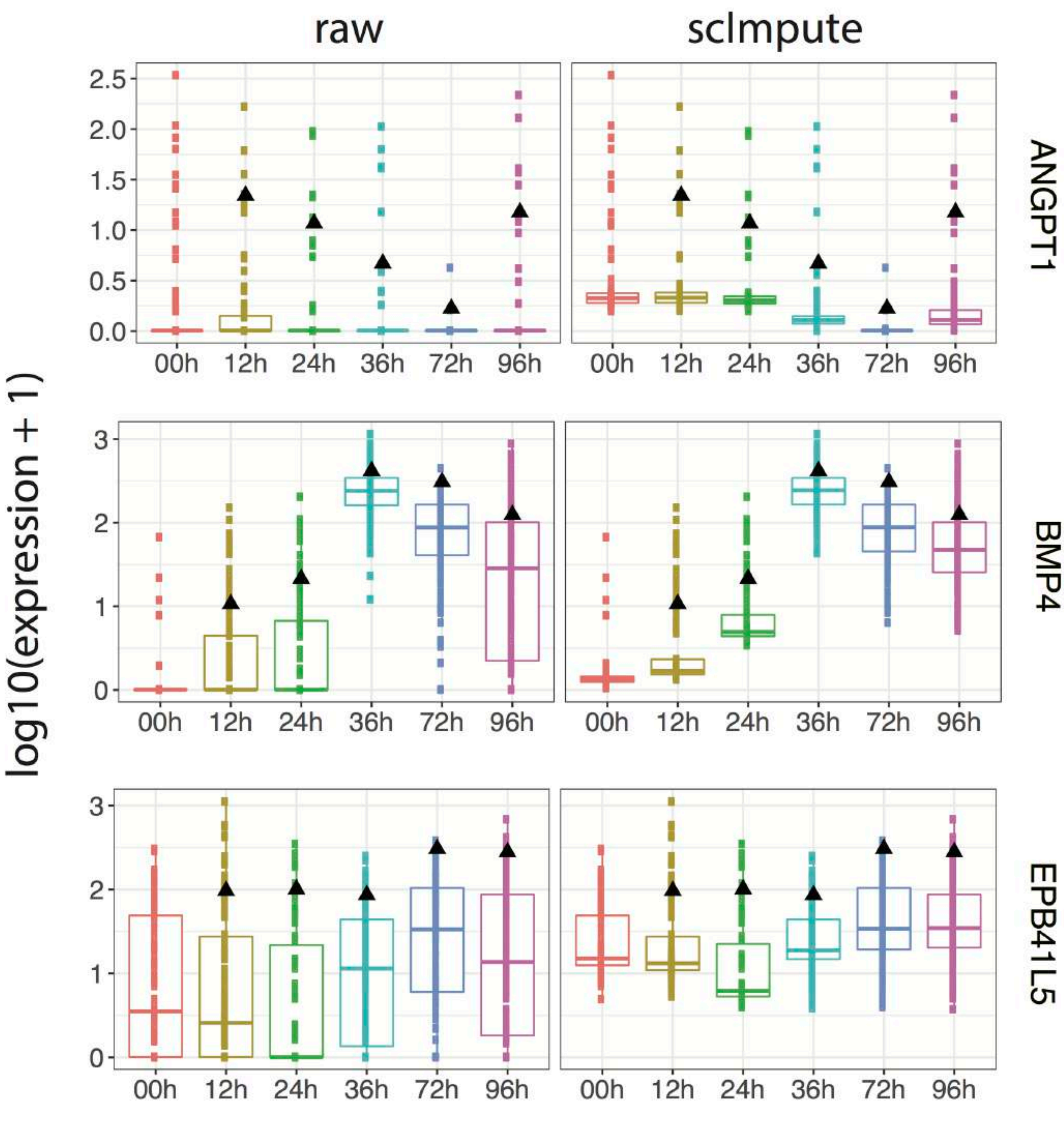

Supplementary Figure 16. Time-course expression patterns of three example genes that are annotated with GO term "endoderm development."

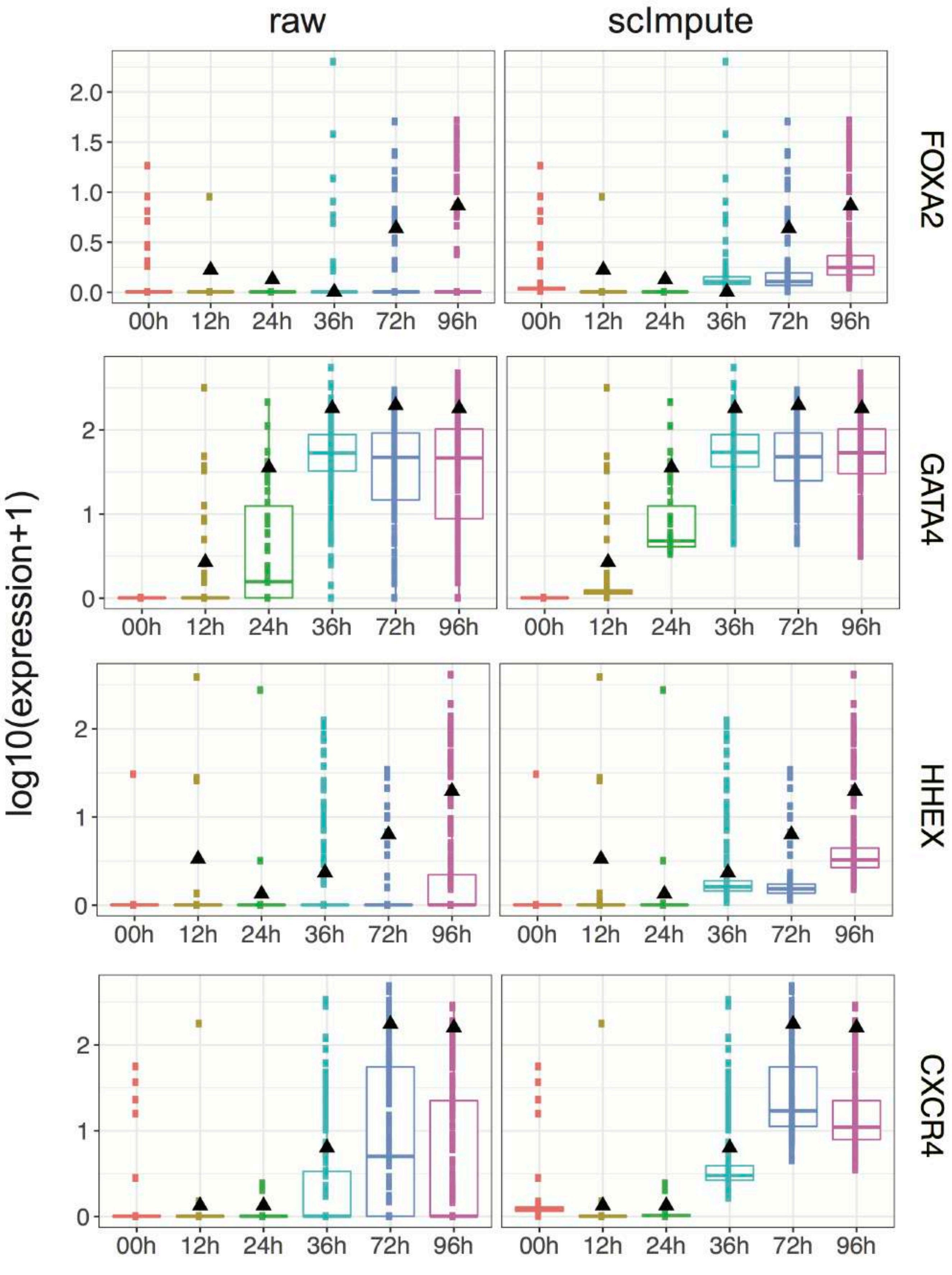

Supplementary Figure 17. Time-course expression patterns of four marker genes of DECs.

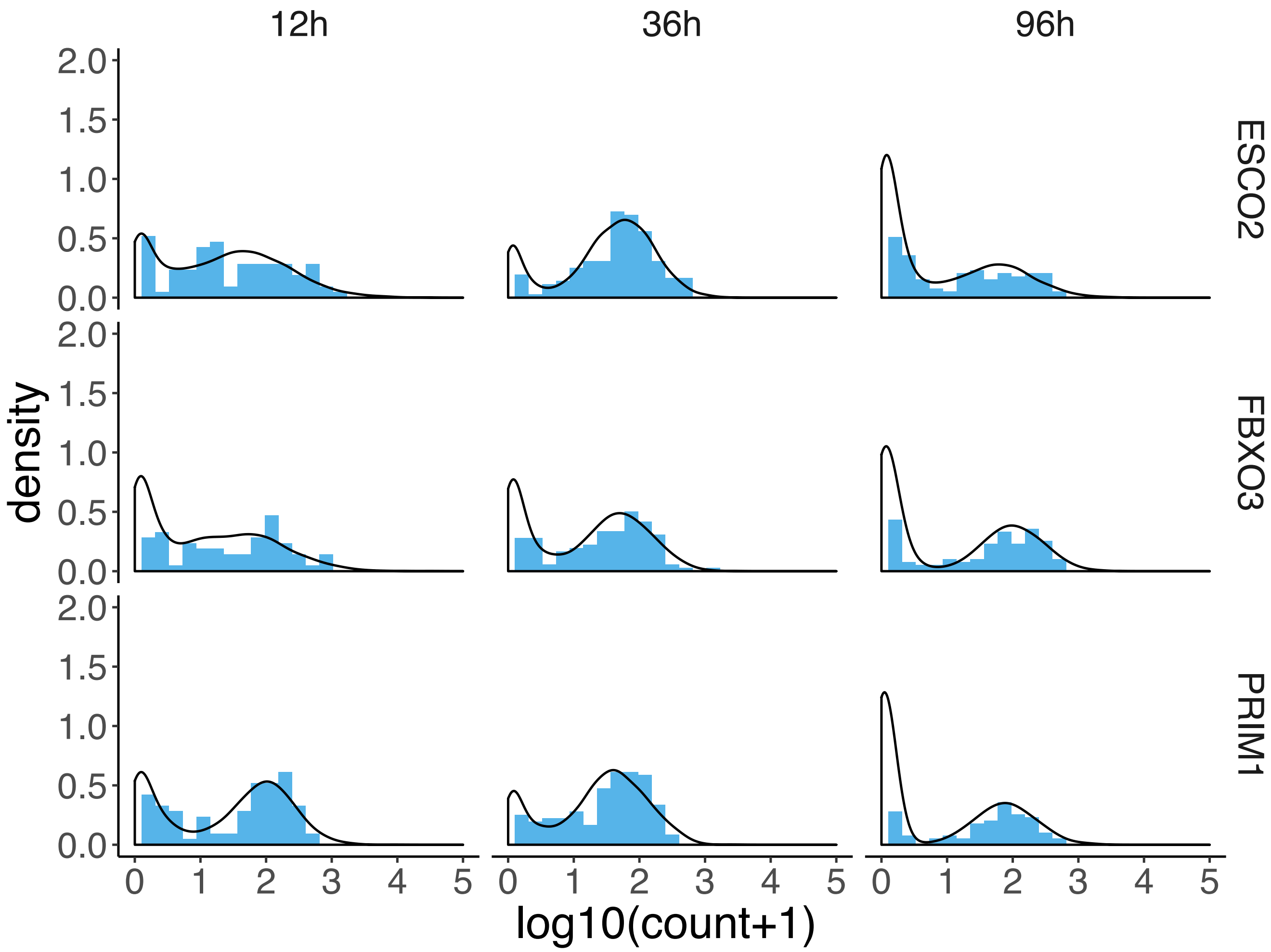

Supplementary Figure 18. Three example genes (*ESCO2*, *FBXO3*, and *PRIM1*) for comparison of observed and fitted expression distribution in three different cell types. The results are based on the human ESC data. Blue histograms represent observed distributions, and black lines represent fitted distributions by the Gamma-Gaussian mixture model (Equation (1)).

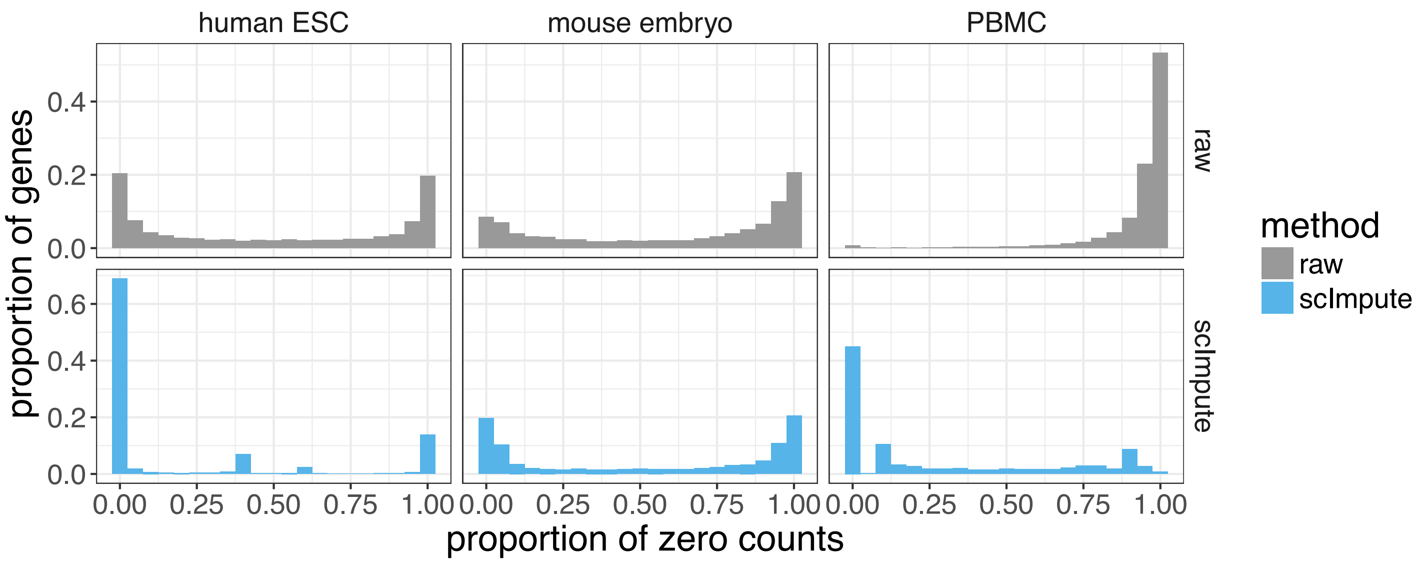

Supplementary Figure 19. Three examples in real data showing the proportions of genes with various proportions of zero expression values across cells in the raw and imputed data. The proportions of zero values in the imputed data are significantly reduced compared with the raw data.

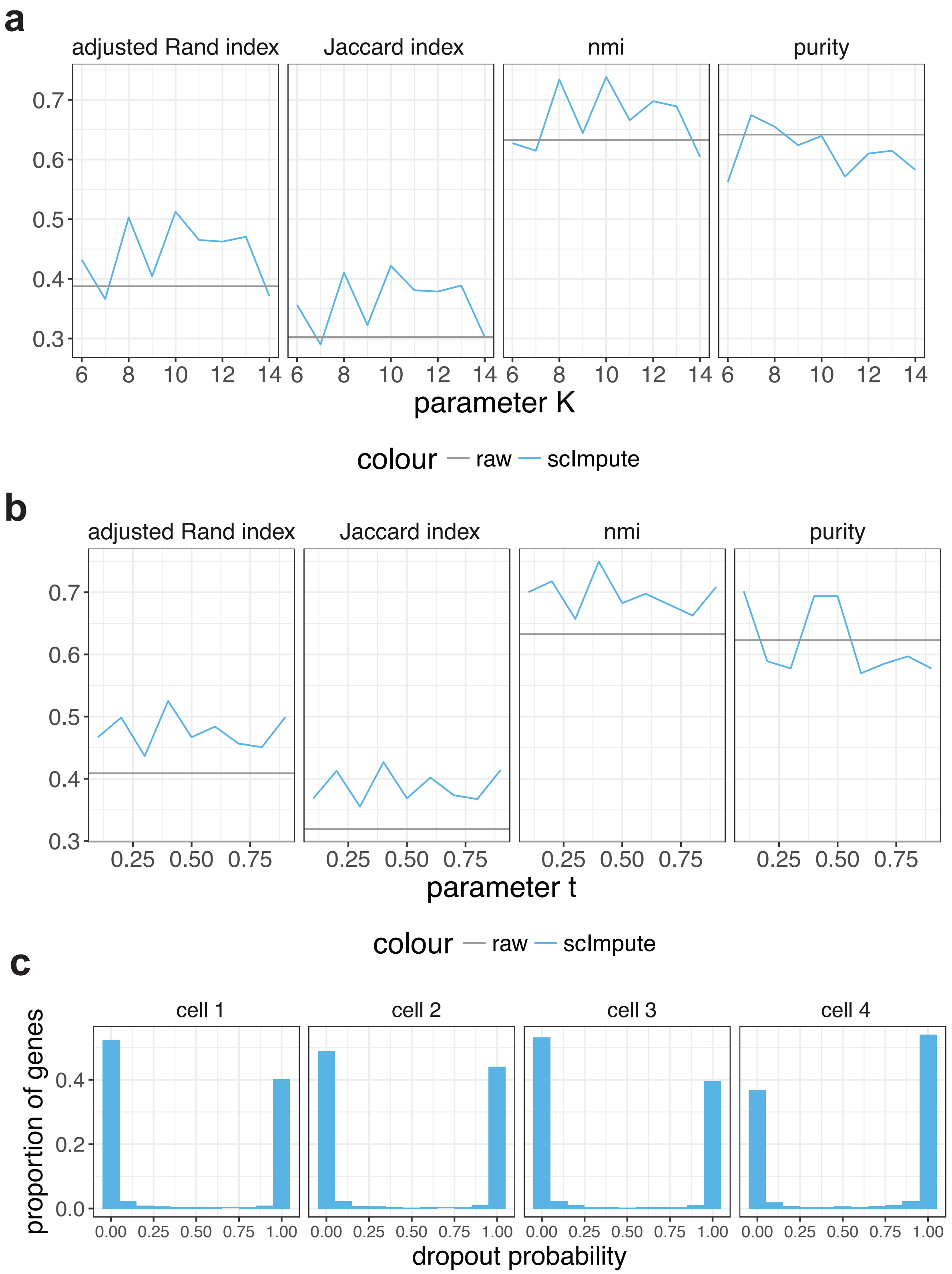

Supplementary Figure 20. Sensitivity analysis based on the mouse embryo data. a: Clustering results of imputed data when different values of parameter K are used in scImpute. **b**: Clustering results of imputed data when different values of parameter t are used in scImpute. **c**: The distribution of dropout probabilities in four randomly selected cells from the mouse embryo data. Most genes have dropout probabilities very close to either 0 or 1.

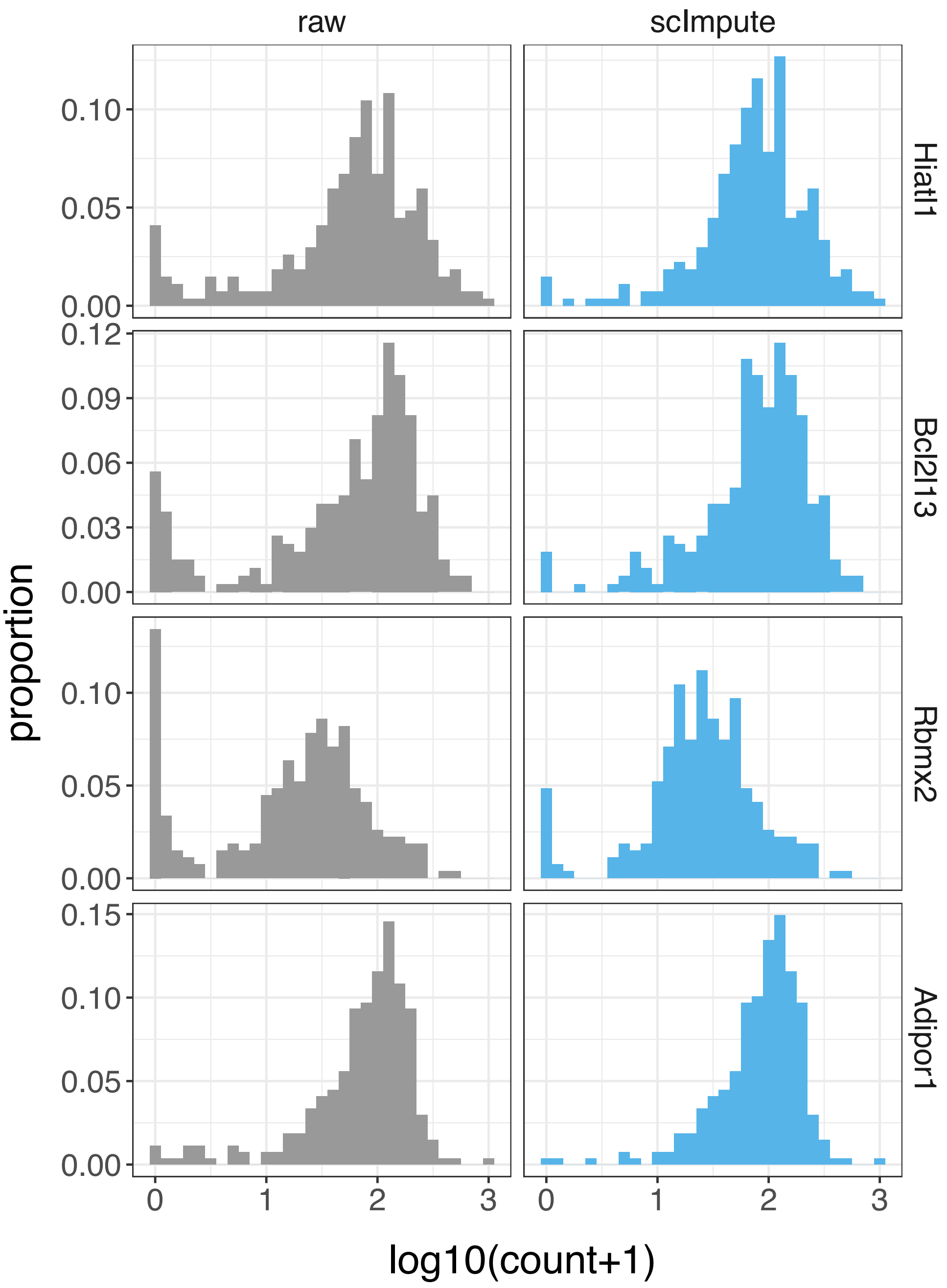

Supplementary Figure 21. The distributions of four examples genes' expression levels across cells before and after imputation. The first column gives the distributions in the raw data, and the second column gives the distributions in the imputed data by scImpute.

Supplementary Tables

		clustered classes		
		pairs in the same class	pairs in different classes	total
true classes	pairs in the same class	a	c	$a + c$
	pairs in different classes	b	d	$b + d$
		$a + b$	$c + d$	

Supplementary Table 1. Confusion matrix used to calculate the clustering measures.

time point	00h	12h	24h	36h	72h	96h	total
scRNA-seq (cells)	92	102	66	172	138	188	758
bulk RNA-seq (replicates)	0	3	3	3	3	3	15

Supplementary Table 2. Sample information of the timecourse RNA-seq data.

data	reference	% zero counts in raw data	% zero counts in imputed data	platform
ERCC spike-ins	19	27.1	0.1	Fluidigm C1
cell cycle	20	22.6	0.5	Fluidigm C1
mouse embryo	22	61.0	52.2	customized
PBMC	10	98.6	28.3	10x Genomics
H1 vs. DEC	30	49.1	20.9	Fluidigm C1
H1 vs. DEC (time-course)	30	54.6	23.6	Fluidigm C1

Supplementary Table 3. Summary of scRNA-seq data used in this manuscript.

GO Term	Description	P-value	GO Term	Description	P-value
GO:0048646	anatomical structure formation involved in morphogenesis	4.86E-11	GO:1902680	positive regulation of RNA biosynthetic process	1.44E-04
GO:0032502	developmental process	2.53E-09	GO:0070169	positive regulation of biomineral tissue development	1.60E-04
GO:2000026	regulation of multicellular organismal development	9.38E-09	GO:0030198	extracellular matrix organization	1.62E-04
GO:0045597	positive regulation of cell differentiation	2.51E-08	GO:0001837	epithelial to mesenchymal transition	1.64E-04
GO:0045595	regulation of cell differentiation	3.38E-08	GO:0043062	extracellular structure organization	1.67E-04
GO:0050793	regulation of developmental process	4.96E-08	GO:0060363	cranial suture morphogenesis	1.71E-04
GO:0051094	positive regulation of developmental process	9.31E-08	GO:0048562	embryonic organ morphogenesis	1.72E-04
GO:0051239	regulation of multicellular organismal process	2.46E-07	GO:0051241	negative regulation of multicellular organismal process	1.85E-04
GO:0048856	anatomical structure development	2.81E-07	GO:0045944	positive regulation of transcription from RNA polymerase II promoter	1.90E-04
GO:0048869	cellular developmental process	3.60E-07	GO:0010646	regulation of cell communication	1.98E-04
GO:0030154	cell differentiation	3.91E-07	GO:0042221	response to chemical	2.14E-04
GO:0048513	animal organ development	5.47E-07	GO:0006928	movement of cell or subcellular component	2.22E-04
GO:0071310	cellular response to organic substance	6.98E-07	GO:0022603	regulation of anatomical structure morphogenesis	2.69E-04
GO:0098609	cell-cell adhesion	8.42E-07	GO:0051093	negative regulation of developmental process	2.83E-04
GO:0051240	positive regulation of multicellular organismal process	8.54E-07	GO:0023051	regulation of signaling	2.88E-04
GO:0045778	positive regulation of ossification	1.15E-06	GO:0050679	positive regulation of epithelial cell proliferation	2.96E-04
GO:0001525	angiogenesis	1.21E-06	GO:0048523	negative regulation of cellular process	3.00E-04
GO:0070887	cellular response to chemical stimulus	2.00E-06	GO:0007568	aging	3.22E-04
GO:0048729	tissue morphogenesis	3.18E-06	GO:0022407	regulation of cell-cell adhesion	3.23E-04
GO:0070848	response to growth factor	4.21E-06	GO:0042482	positive regulation of odontogenesis	3.29E-04
GO:0048598	embryonic morphogenesis	5.49E-06	GO:0007178	transmembrane receptor protein serine/threonine kinase signaling pathway	3.39E-04
GO:0071560	cellular response to transforming growth factor beta stimulus	5.96E-06	GO:0048732	gland development	3.45E-04
GO:0045669	positive regulation of osteoblast differentiation	8.61E-06	GO:0048585	negative regulation of response to stimulus	3.48E-04
GO:0009653	anatomical structure morphogenesis	1.21E-05	GO:0051254	positive regulation of RNA metabolic process	3.61E-04
GO:0003198	epithelial to mesenchymal transition involved in endocardial cushion formation	1.26E-05	GO:0060317	cardiac epithelial to mesenchymal transition	3.83E-04
GO:0071495	cellular response to endogenous stimulus	1.29E-05	GO:0030155	regulation of cell adhesion	3.90E-04
GO:0061138	morphogenesis of a branching epithelium	1.47E-05	GO:1903707	negative regulation of hemopoiesis	4.03E-04
GO:0071559	response to transforming growth factor beta	1.52E-05	GO:0035239	tube morphogenesis	4.14E-04
GO:0042481	regulation of odontogenesis	1.85E-05	GO:0045165	cell fate commitment	4.23E-04
GO:0010038	response to metal ion	1.99E-05	GO:0048519	negative regulation of biological process	4.34E-04
GO:0071363	cellular response to growth factor stimulus	2.25E-05	GO:0071241	cellular response to inorganic substance	4.38E-04
GO:0030500	regulation of bone mineralization	2.30E-05	GO:0043392	negative regulation of DNA binding	4.63E-04
GO:0030278	regulation of ossification	2.39E-05	GO:1904381	Golgi apparatus mannose trimming	4.98E-04
GO:0048754	branching morphogenesis of an epithelial tube	2.50E-05	GO:0060129	thyroid-stimulating hormone-secreting cell differentiation	4.98E-04
GO:0001763	morphogenesis of a branching structure	2.52E-05	GO:0051270	regulation of cellular component movement	5.01E-04
GO:0035116	embryonic hindlimb morphogenesis	2.71E-05	GO:0060429	epithelium development	5.38E-04
GO:2000677	regulation of transcription regulatory region DNA binding	3.08E-05	GO:0014070	response to organic cyclic compound	5.44E-04
GO:0048583	regulation of response to stimulus	3.43E-05	GO:0061029	eyelid development in camera-type eye	5.60E-04
GO:0048518	positive regulation of biological process	3.45E-05	GO:0048762	mesenchymal cell differentiation	6.03E-04
GO:0048468	cell development	3.83E-05	GO:0071407	cellular response to organic cyclic compound	6.14E-04
GO:0007166	cell surface receptor signaling pathway	4.11E-05	GO:0003006	developmental process involved in reproduction	6.38E-04
GO:0009966	regulation of signal transduction	4.46E-05	GO:0030326	embryonic limb morphogenesis	6.45E-04
GO:0035115	embryonic forelimb morphogenesis	4.54E-05	GO:0035113	embryonic appendage morphogenesis	6.45E-04
GO:0045785	positive regulation of cell adhesion	4.68E-05	GO:0051101	regulation of DNA binding	6.46E-04
GO:0070167	regulation of biomineral tissue development	5.30E-05	GO:1904018	positive regulation of vasculature development	6.76E-04
GO:0048522	positive regulation of cellular process	5.64E-05	GO:0008284	positive regulation of cell proliferation	6.84E-04
GO:0010033	response to organic substance	6.09E-05	GO:2001212	regulation of vasculogenesis	7.06E-04
GO:0002009	morphogenesis of an epithelium	7.04E-05	GO:0071248	cellular response to metal ion	7.06E-04
GO:0009887	animal organ morphogenesis	7.67E-05	GO:0007165	signal transduction	7.11E-04
GO:0030501	positive regulation of bone mineralization	8.32E-05	GO:0030855	epithelial cell differentiation	7.21E-04
GO:0032501	multicellular organismal process	8.47E-05	GO:0048565	digestive tract development	7.22E-04
GO:0045667	regulation of osteoblast differentiation	8.77E-05	GO:0045596	negative regulation of cell differentiation	7.24E-04
GO:0035137	hindlimb morphogenesis	9.56E-05	GO:0002683	negative regulation of immune system process	8.04E-04
GO:0035295	tube development	9.85E-05	GO:0042127	regulation of cell proliferation	8.36E-04
GO:1902105	regulation of leukocyte differentiation	1.08E-04	GO:0030203	glycosaminoglycan metabolic process	8.78E-04
GO:0007155	cell adhesion	1.10E-04	GO:0051592	response to calcium ion	8.80E-04
GO:0035584	calcium-mediated signaling using intracellular calcium source	1.14E-04	GO:0035148	tube formation	8.80E-04

Supplementary Table 4. Go enrichment analysis. Enriched GO terms ($p < 10^{-3}$) in the 244 DEC up-regulated genes that are only detected in scImpute's imputed data by DESeq2 [31]. The texts marked in red are the GO terms related to DEC functions.

GO Term	Description	P-value	GO Term	Description	P-value
GO:0032502	developmental process	7.42E-12	GO:0051252	regulation of RNA metabolic process	1.06E-04
GO:0048856	anatomical structure development	7.59E-12	GO:0042481	regulation of odontogenesis	1.07E-04
GO:0048646	anatomical structure formation involved in morphogenesis	2.99E-11	GO:0009887	animal organ morphogenesis	1.12E-04
GO:0030154	cell differentiation	1.30E-08	GO:0003002	regionalization	1.12E-04
GO:0050793	regulation of developmental process	2.21E-08	GO:0072073	kidney epithelium development	1.13E-04
GO:0009653	anatomical structure morphogenesis	2.47E-08	GO:0023051	regulation of signaling	1.22E-04
GO:0035116	embryonic hindlimb morphogenesis	3.14E-08	GO:0061312	BMP signaling pathway involved in heart development	1.25E-04
GO:0048869	cellular developmental process	4.97E-08	GO:0061180	mammary gland epithelium development	1.25E-04
GO:2000026	regulation of multicellular organismal development	7.95E-08	GO:0048762	mesenchymal cell differentiation	1.26E-04
GO:0060411	cardiac septum morphogenesis	1.81E-07	GO:0010646	regulation of cell communication	1.26E-04
GO:0051094	positive regulation of developmental process	2.06E-07	GO:0001958	endochondral ossification	1.29E-04
GO:0035137	hindlimb morphogenesis	2.69E-07	GO:0036075	replacement ossification	1.29E-04
GO:0048513	animal organ development	3.45E-07	GO:0060317	cardiac epithelial to mesenchymal transition	1.29E-04
GO:0051093	negative regulation of developmental process	5.96E-07	GO:0001568	blood vessel development	1.48E-04
GO:0032501	multicellular organismal process	8.84E-07	GO:0048523	negative regulation of cellular process	1.53E-04
GO:0022603	regulation of anatomical structure morphogenesis	1.47E-06	GO:0060349	bone morphogenesis	1.55E-04
GO:0003198	epithelial to mesenchymal transition involved in endocardial	1.60E-06	GO:0045596	negative regulation of cell differentiation	1.99E-04
GO:0098609	cell-cell adhesion	1.98E-06	GO:0045892	negative regulation of transcription, DNA-templated	2.01E-04
GO:0048598	embryonic morphogenesis	2.32E-06	GO:1903507	negative regulation of nucleic acid-templated transcription	2.12E-04
GO:0006357	regulation of transcription from RNA polymerase II promoter	2.65E-06	GO:0003179	heart valve morphogenesis	2.18E-04
GO:0060429	epithelium development	2.78E-06	GO:2000241	regulation of reproductive process	2.19E-04
GO:0048729	tissue morphogenesis	2.80E-06	GO:1902679	negative regulation of RNA biosynthetic process	2.19E-04
GO:0045893	positive regulation of transcription, DNA-templated	3.77E-06	GO:0030500	regulation of bone mineralization	2.30E-04
GO:1903508	positive regulation of nucleic acid-templated transcription	3.77E-06	GO:0030512	negative regulation of transforming growth factor beta receptor	2.30E-04
GO:1902680	positive regulation of RNA biosynthetic process	3.85E-06	GO:2000677	regulation of transcription regulatory region DNA binding	2.33E-04
GO:0051239	regulation of multicellular organismal process	4.30E-06	GO:0035904	aorta development	2.38E-04
GO:0001763	morphogenesis of a branching structure	4.75E-06	GO:0048583	regulation of response to stimulus	2.42E-04
GO:0045595	regulation of cell differentiation	5.27E-06	GO:0002009	morphogenesis of an epithelium	2.46E-04
GO:0003148	outflow tract septum morphogenesis	5.44E-06	GO:0001667	ameboidal-type cell migration	2.47E-04
GO:0001525	angiogenesis	6.50E-06	GO:0051173	positive regulation of nitrogen compound metabolic process	2.49E-04
GO:0051254	positive regulation of RNA metabolic process	7.01E-06	GO:0007411	axon guidance	2.74E-04
GO:0048522	positive regulation of cellular process	7.62E-06	GO:1903845	negative regulation of cellular response to transforming growth	2.78E-04
GO:0009888	tissue development	8.20E-06	GO:0031325	positive regulation of cellular metabolic process	3.00E-04
GO:0035239	tube morphogenesis	9.92E-06	GO:0097094	craniofacial suture morphogenesis	3.01E-04
GO:0007155	cell adhesion	1.02E-05	GO:0098742	cell-cell adhesion via plasma-membrane adhesion molecules	3.12E-04
GO:0090092	regulation of transmembrane receptor protein serine signaling	1.05E-05	GO:0009966	regulation of signal transduction	3.13E-04
GO:0022610	biological adhesion	1.15E-05	GO:0001503	ossification	3.16E-04
GO:0061138	morphogenesis of a branching epithelium	1.32E-05	GO:0048589	developmental growth	3.21E-04
GO:0090287	regulation of cellular response to growth factor stimulus	1.34E-05	GO:0071657	positive regulation of granulocyte colony-stimulating production	3.50E-04
GO:0006355	regulation of transcription, DNA-templated	1.46E-05	GO:2001055	positive regulation of mesenchymal cell apoptotic process	3.50E-04
GO:0048754	branching morphogenesis of an epithelial tube	1.48E-05	GO:1901258	positive regulation of macrophage colony-stimulating factor	3.50E-04
GO:0017015	regulation of transforming growth factor beta receptor signaling	1.67E-05	GO:0051147	regulation of muscle cell differentiation	3.54E-04
GO:0006928	movement of cell or subcellular component	1.69E-05	GO:0042127	regulation of cell proliferation	3.55E-04
GO:1903506	regulation of nucleic acid-templated transcription	1.92E-05	GO:0035148	tube formation	3.83E-04
GO:1903844	regulation of cellular response to transforming growth factor	1.99E-05	GO:0040007	growth	3.84E-04
GO:0030326	embryonic limb morphogenesis	2.05E-05	GO:0001822	kidney development	4.07E-04
GO:0035113	embryonic appendage morphogenesis	2.05E-05	GO:0051253	negative regulation of RNA metabolic process	4.28E-04
GO:0035115	embryonic forelimb morphogenesis	2.06E-05	GO:0048562	embryonic organ morphogenesis	4.33E-04
GO:2001141	regulation of RNA biosynthetic process	2.13E-05	GO:0023019	signal transduction involved in regulation of gene expression	4.63E-04
GO:0051240	positive regulation of multicellular organismal process	2.56E-05	GO:0048519	negative regulation of biological process	4.76E-04
GO:0010628	positive regulation of gene expression	2.71E-05	GO:0035295	tube development	4.76E-04
GO:0048518	positive regulation of biological process	2.90E-05	GO:0009893	positive regulation of metabolic process	4.89E-04
GO:0009891	positive regulation of biosynthetic process	3.55E-05	GO:2000243	positive regulation of reproductive process	5.02E-04
GO:2000678	negative regulation of transcription regulatory DNA binding	3.57E-05	GO:0003139	secondary heart field specification	5.04E-04
GO:0061311	cell surface receptor signaling pathway involved in heart	3.57E-05	GO:0003128	heart field specification	5.04E-04
GO:0045597	positive regulation of cell differentiation	3.71E-05	GO:0060363	cranial suture morphogenesis	5.04E-04
GO:0043392	negative regulation of DNA binding	3.82E-05	GO:0070167	regulation of biomineral tissue development	5.09E-04
GO:0051270	regulation of cellular component movement	3.87E-05	GO:0060560	developmental growth involved in morphogenesis	5.10E-04
GO:0045944	positive regulation of transcription from RNA polymerase II	8.08E-05	GO:0008283	cell proliferation	6.73E-04
GO:0097485	neuron projection guidance	8.08E-05	GO:0001657	ureteric bud development	6.78E-04
GO:0042692	muscle cell differentiation	8.62E-05	GO:0007369	gastrulation	6.78E-04

Supplementary Table 5. Go enrichment analysis. Enriched GO terms ($p < 10^{-3}$) in the 339 DEC up-regulated genes that are only detected in scImpute's imputed data by MAST [32]. The texts marked in red are the GO terms related to DEC functions.

GO Term	Description	P-value
GO:0018401	peptidyl-proline hydroxylation to 4-hydroxy-L-proline	5.97E-05
GO:0030029	actin filament-based process	1.58E-04
GO:0065007	biological regulation	1.61E-04
GO:0034113	heterotypic cell-cell adhesion	1.67E-04
GO:0030036	actin cytoskeleton organization	2.30E-04
GO:1903829	positive regulation of cellular protein localization	3.29E-04
GO:0034446	substrate adhesion-dependent cell spreading	6.18E-04
GO:0065008	regulation of biological quality	6.25E-04
GO:0048583	regulation of response to stimulus	6.79E-04

Supplementary Table 6. Go enrichment analysis. Enriched GO terms ($p < 10^{-3}$) in the 249 DEC up-regulated genes that are only detected in raw data by DESeq2 [31].

GO Term	Description	P-value	GO Term	Description	P-value
GO:0071840	cellular component organization or biogenesis	2.69E-08	GO:0071310	cellular response to organic substance	2.30E-04
GO:0006260	DNA replication	7.56E-08	GO:0044806	G-quadruplex DNA unwinding	2.33E-04
GO:0016043	cellular component organization	8.79E-08	GO:0120035	regulation of plasma membrane bounded cell projection organization	2.36E-04
GO:0032508	DNA duplex unwinding	1.14E-06	GO:0033627	cell adhesion mediated by integrin	2.63E-04
GO:0048869	cellular developmental process	2.57E-06	GO:0030334	regulation of cell migration	2.66E-04
GO:0071897	DNA biosynthetic process	2.67E-06	GO:0048518	positive regulation of biological process	2.86E-04
GO:0022604	regulation of cell morphogenesis	3.23E-06	GO:0070252	actin-mediated cell contraction	3.01E-04
GO:0032392	DNA geometric change	3.82E-06	GO:0031344	regulation of cell projection organization	3.05E-04
GO:0001649	osteoblast differentiation	3.87E-06	GO:0009987	cellular process	3.19E-04
GO:0048523	negative regulation of cellular process	6.03E-06	GO:0030048	actin filament-based movement	3.25E-04
GO:0048519	negative regulation of biological process	6.05E-06	GO:0022616	DNA strand elongation	3.33E-04
GO:0030198	extracellular matrix organization	6.38E-06	GO:0070887	cellular response to chemical stimulus	3.38E-04
GO:0032502	developmental process	6.68E-06	GO:0009719	response to endogenous stimulus	3.48E-04
GO:0043062	extracellular structure organization	6.69E-06	GO:0034330	cell junction organization	3.90E-04
GO:0043504	mitochondrial DNA repair	7.06E-06	GO:0050767	regulation of neurogenesis	4.03E-04
GO:0000732	strand displacement	8.07E-06	GO:0008285	negative regulation of cell proliferation	4.06E-04
GO:0042127	regulation of cell proliferation	1.26E-05	GO:0007010	cytoskeleton organization	4.42E-04
GO:0035987	endodermal cell differentiation	1.28E-05	GO:0000904	cell morphogenesis involved in differentiation	4.58E-04
GO:0030516	regulation of axon extension	1.49E-05	GO:0008284	positive regulation of cell proliferation	4.77E-04
GO:0007049	cell cycle	1.93E-05	GO:0051093	negative regulation of developmental process	4.77E-04
GO:0022610	biological adhesion	2.01E-05	GO:0022607	cellular component assembly	4.90E-04
GO:0010975	regulation of neuron projection development	2.08E-05	GO:0010769	regulation of cell morphogenesis involved in differentiation	4.99E-04
GO:0071103	DNA conformation change	2.78E-05	GO:0051276	chromosome organization	5.04E-04
GO:0051716	cellular response to stimulus	2.99E-05	GO:0006928	movement of cell or subcellular component	5.06E-04
GO:1901796	regulation of signal transduction by p53 class	3.53E-05	GO:0050793	regulation of developmental process	5.19E-04
GO:0060284	regulation of cell development	3.66E-05	GO:1901566	organonitrogen compound biosynthetic process	5.68E-04
GO:0010811	positive regulation of cell-substrate adhesion	4.11E-05	GO:0045664	regulation of neuron differentiation	5.87E-04
GO:0007155	cell adhesion	4.23E-05	GO:1903047	mitotic cell cycle process	6.44E-04
GO:0048513	animal organ development	4.37E-05	GO:0090100	positive regulation of transmembrane receptor protein serine/threonine	6.48E-04
GO:0051052	regulation of DNA metabolic process	5.68E-05	GO:0048583	regulation of response to stimulus	6.52E-04
GO:0061387	regulation of extent of cell growth	5.84E-05	GO:0051128	regulation of cellular component organization	6.61E-04
GO:0010810	regulation of cell-substrate adhesion	5.94E-05	GO:0051270	regulation of cellular component movement	6.92E-04
GO:0006259	DNA metabolic process	8.21E-05	GO:0040012	regulation of locomotion	7.05E-04
GO:0000731	DNA synthesis involved in DNA repair	8.64E-05	GO:0010033	response to organic substance	7.25E-04
GO:0030029	actin filament-based process	8.93E-05	GO:0050794	regulation of cellular process	7.28E-04
GO:0042493	response to drug	1.08E-04	GO:0009611	response to wounding	7.48E-04
GO:0010721	negative regulation of cell development	1.16E-04	GO:0034446	substrate adhesion-dependent cell spreading	7.63E-04
GO:0097435	supramolecular fiber organization	1.25E-04	GO:0030049	muscle filament sliding	7.63E-04
GO:0042221	response to chemical	1.50E-04	GO:0033275	actin-myosin filament sliding	7.63E-04
GO:2000145	regulation of cell motility	1.73E-04	GO:0010977	negative regulation of neuron projection development	7.88E-04
GO:0050678	regulation of epithelial cell proliferation	1.74E-04	GO:0023051	regulation of signaling	7.95E-04
GO:0048856	anatomical structure development	1.77E-04	GO:0010646	regulation of cell communication	8.19E-04
GO:0022603	regulation of anatomical structure	1.81E-04	GO:0043687	post-translational protein modification	8.58E-04
GO:0050770	regulation of axonogenesis	1.98E-04	GO:0045773	positive regulation of axon extension	9.69E-04
GO:0030154	cell differentiation	2.03E-04	GO:0007044	cell-substrate junction assembly	9.69E-04
GO:0051782	negative regulation of cell division	2.04E-04	GO:0007420	brain development	9.79E-04
GO:0043200	response to amino acid	2.08E-04			

Supplementary Table 7. Go enrichment analysis. Enriched GO terms ($p < 10^{-3}$) in the 339 DEC up-regulated genes that are only detected in the raw data by MAST [32]. The texts marked in red are the GO terms related to DEC functions.
